# Appendix to the Clinical Lecture on Aphonia, Etc.

**Published:** 1848-12

**Authors:** 


*Appendix to the Clinical Lecture on Aphonia, etc.*—By Prof. Lee. *Case of Aphonia.*

Since the clinical lecture on aphonia and stammering, contained in the department of original communications of this number, was in type, Dr. Lee has forwarded the following note, giving an account of another interesting case of aphonia, which we introduce here, as an appendix to his valuable article:—

A very singular case of loss of speech, somewhat resembling the above, has recently been presented at our Clinique. M. C., a little girl, three years of age; was bitten in the cheek when twenty months old, by a rabid cat. The wound healed speedily, but about five weeks from the occurrence of the accident, violent spasms came on, affecting nearly all the muscles of the body, and which continued at longer or shorter intervals from Sunday until Friday evening following. The spasms then ceased and she gradually got better; but it was soon ascertained that she had lost the power of speech, for she had begun to talk considerably, and also the use of her limbs. The parents state that during the hydrophobic attack, she was several times so far reduced as to be supposed to be dead, and was actually laid out once or twice, when she recovered. She now makes a few harsh sounds, like the deaf and dumb, but can not articulate. Her hearing is quick; her face is not wanting in intelligence; but she is remarkably susceptible to bright lights, and sounds; constantly in active motion, and little inclined to sleep, it often requiring two or three hours to calm her to rest. She has a constant propensity to stand on her head and turn somersets, and she is employed a great deal of the time in such feats, traversing the room in a succession of somersets, with astonishing rapidity. If left alone for a few minutes she is often found standing in her head in the rocking chair, her hands resting on the arms of the chair, while her feet are thrown up against the back of the same. The top of the head is preternaturally hot over a space of two or three square inches; her pupils are much of the time considerably dilated. Her appetite is good, as is also her general health.

The only rational explanation of the above case, appears to me to be that which attributes the loss of speech, and the other symptoms, to cerebral derangement, consequent upon the disease which caused the spasms, &c. Some organic change has occurred, incompatible with the normal exercise of the intellectual faculties, and reducing her to a state bordering on idiocy. The case is therefore, probably, a hopeless one; though it is impossible to predict with certainty, what will be the result. Medical treatment, however, promises but little.

